# As if I was a spacecraft returning to Earth’s atmosphere. Expanding insights into illness narratives and childhood cancer through evocative autoethnography

**DOI:** 10.1177/13634593231200123

**Published:** 2023-09-20

**Authors:** Eva-Mari Andersen

**Affiliations:** University of Oslo, Norway

**Keywords:** Arthur Frank, autoethnography, childhood cancer, illness narrative, school re-entry

## Abstract

Today, a majority of children diagnosed with cancer are expected to grow up and live—hopefully until old age. Still, knowledge of the lived experience of childhood cancer survivors is sparse. In pursuit of knowledge expansion, by combining my intersecting roles as an academic, educational counselor, and childhood cancer survivor, I approach my personal illness narrative. By means of evocative autoethnography, I write intentionally vulnerably about my experiences and make them available for consideration. I explore my narrative through archives, artifacts, memories of the past, and conversations evoked in the present. I re-visit the cultural landscape of a southern Norwegian girl growing up in the 00s with cancer. Through this, my illness narrative presents as positioned, tangled, and interwoven with a developmental trajectory. Specific educational experiences seem to linger, and many are related to being absent from or re-entering school after the onset of illness. To grasp the intersecting and conflicting experiences of being very ill while also young, I suggest Erik Erikson’s moratorium as a key concept. To complement Arthur Frank’s illness narratives of restitution, chaos, and quest, I establish the moratorium narrative. As a fresh resource, the moratorium narrative underlines the need to make sensitive our academic community’s gaze on illness trajectories unfolding in formative phases and illness narratives defined by growing up. By providing a point of recognition that prompts elaboration, this could also provide the young and very ill with a much-needed narrative space of opportunity, of which more narratives are invited and insisted upon.

## The narrative of childhood cancer

The recorded modern history of childhood cancer is brief, unfolding from the 1940s to today (Mukherjee, 2010). In Norway, the year 1954 is reported as a benchmark in this history as it was when systematic treatment with chemotherapy was introduced (Lie, 1993). With the introduction of chemotherapy, the care of children with cancer changed—from focusing primarily on terminal care to curation. Today, a majority of children diagnosed with cancer are expected to grow up and live—hopefully until old age. Thus, core to the narrative of childhood cancer is modern medical advancements. Nevertheless, although the fate of childhood cancer patients has changed, their lived experience still revolve around prolonged and intrusive treatment regimens, isolation, pain, discomfort, and despair.

To young cancer patients, absence from school is one of several burdens. While isolated from their everyday arenas, young patients emphasize the importance of keeping in touch with peers and share mixed feelings, both anticipation and anxiety, toward returning to them (Wilkie, 2012; Yi et al., 2016). After a prolonged absence, leaving the hospital to re-enter a local, regular school setting is considered a pivotal milestone (McLoone et al., 2013). Although unmet educational needs are commonly reported in this vulnerable process, teachers represent potential protective influences (Ferguson and Walker, 2014; Hocking et al., 2018; Robinson and Summers, 2012). Less communicated in the narrative of childhood cancer and rarely in the focus of educational research, these dimensions of experience are timely to address.

Growing up is a complex process involving the pursuit of physical, personal, and cognitive milestones (Woolfolk, 2016). In Erik Erikson’s influential, though contested, theory of psychosocial development (Erikson, 1968, 1980; Schwartz et al., 2018), this is described as a series of transitions and conflicts that define an emerging sense of self. In Erikson’s terminology, the undecided length of time that must be set aside for considering and adjusting to identity-related issues is conceptualized as moratorium. Linked to the youthful experience of being delayed, in-between, and in transition, moratorium is considered a key concept in this article.

As young cancer patients experience intersecting struggles related to growing up and suffering from a severe, potentially deadly disease, their embodied experience conflicts with developmental timelines. This may involve feelings of growing up faster and being more mature than peers but also becoming more dependent when independence is expected (Dattilo et al., 2021; Kent et al., 2012). Although moratorium has been suggested to accommodate experiences like these, and the time span set to (re)adjust and (re)establish a sense of self after extraordinary life events (Stewart, 1978), the term liminality is more often used in efforts to understand cancer experience (Little et al., 1998). This term, perceived as fundamental to understanding “the changing and the experiencing body housing both the disease and the self” (p. 1485), does not fully capture the way in which the normative developmental timelines of childhood and youth influence the illness experience. While prior work in narrative, autoethnography, and pathography sheds light on the harsh reality of simultaneously navigating illness and being young (see, e.g. Andersen, 2022; Hannum, 2017; Jaouad, 2021), they have yet to be fully utilized in analyzing theories related to illness and narratives. By means of my personal illness narrative and the concept of moratorium, I will, in this article, try to do just this.

In the year of 2000, at age 13, I was diagnosed with high-risk acute lymphoblastic leukemia. As survival statistics steadily climbed, although my prognosis was very grim, I remember this as a time of high hopes. The treatment I was provided was considered state-of-the-art. Eventually, it cured my cancer, but it also wrecked my body. Unexpectedly, it projected me into a different reality for which I was unprepared but gradually learned to accept. To describe my cancer survival today, I find the southern Norwegian landscape I grew up in, with its sleek uphill and uneven shrub forest, a good fit. You must vigilantly watch your step, but navigation is possible. I am now 36, an educational counselor, and an early-career academic. As I write this article, I have been surviving cancer for over two decades. During work hours, I research narratives of young cancer survival and interview young adult cancer survivors about their everyday lives. In my free time, I balance my roles, as mother, partner, daughter, sister, and friend—and still navigate the shrub forest. The duality of interpreting others’ illness narratives while holding one myself is this article’s starting point.

## Prompts to break the silence

Although the position and potential of illness narratives are disputed (see, e.g. Atkinson, 1997; Frank, 2000; Thomas, 2010), the articulations of the ill are recognized as important to our knowledge of health and illness. However, as Frank (2016) writes of “the ill person as a *narrative subject*, defined by discursive possibilities” (p. 9), I see my illness trajectory as influenced by a discourse of silence. As young cancer survivors are cautious in disclosing cancer-related experiences (see, e.g. Rabin, 2020), I have come to know silence as a relief from friction. By not talking about my cancer, I have experienced being perceived as equal to my peers. By masking my needs, I have been given opportunities. Simultaneously I have also longed for a sense of community and repeatedly searched for illness narratives I could relate to as my own.

In *The Wounded Storyteller*, Frank (2013) describes three culturally shared resources as central to the illness narratives of our times, namely restitution, chaos, and quest. Restitution narratives revolve around restoring health by going through illness. In this, illness serves as an interruption, and health is the norm. Through narratives of “tests and their interpretation, treatments and their possible outcomes, the competence of physicians, and alternative treatments,” restitution stories are told (p. 77). Chaos narratives are centered on the unpredictability of illness, disconnection, and loss of control by telling us “how easily any of us could be sucked under” by it (p. 97). However, through voices of chaos, stories of illness can be reconstructed and given coherent form. Quest narratives “meet suffering head on; they accept illness and seek to *use* it” (p. 115). When illness becomes a quest, it serves as a mission, implying that the ill person has been given something through the illness experience, usually insights that must be passed on to others. In the inquiry of illness, restitution, chaos, and quest are popular interpretive tools. Nevertheless, as Frank (2013) emphasizes articulating illness narratives as processes of discovery, I am encouraged to seek and suggest new complementary conceptions of my own.

Based on these intersecting narrative prompts, I will in this article explore my own illness narrative. To re-story my illness narrative today, I re-visit the cultural landscape of a southern Norwegian girl growing up in the 00s with cancer. Here, I find my experiences to be positioned and tangled (Adams, 2018)—interwoven with a developmental trajectory and as part of others’ narratives. In pursuit, my focus shifts from archives, artifacts, memories of the past, and conversations evoked in the present. While writing, I rely on Holman Jones et al. (2013) descriptions of autoethnography as genre and method, and feel empowered by Ellis (1995, 1999) evocative autoethnography. By “embracing vulnerability with purpose” (Holman Jones et al., 2013: 24), I write about my personal experiences, make them available for consideration, and aim to connect and engage with my readers. With hopes of expanding our insights into the young and the very ill, I also discuss my experiences considering established theoretical frames of thinking. Described as subverting “traditional norms of scholarship” (Holman Jones et al., 2013: 35), the form and logic of autoethnographies often differ from traditional article structures. This text is no exception. In the following, I write my narrative through pages 5 to 12 before purposefully examining it through pages 13 to 16.

## The moment I became aware of myself

By vocation I am an educational counselor. Through my first job, while working closely with students needing medical and pedagogical accommodations, I found my profession and felt at home. Here, I experienced being an asset in a classroom and invested a lot of time and most of my energy to get to know the students and scaffold them during our hours together. Sometimes, I would be so exhausted after work hours that I just went to bed fully clothed. I had been working for about six months when I started to dream about my students. In one (still recurring) dream, one of the students with communication challenges approaches me and talks with a voice I have never heard. I stand mesmerized, both ashamed and humble, while she comments on specific situations from our interactions and evaluates my efforts to understand her. Looking back, I remember how this student evoked extreme emotions in me—love, joy, frustration, and sorrow—challenging my abilities and knowledge. She seems to have made a lasting imprint—in my mind and my professional sense of self.

I have reflected on how the students I have met through my work have shaped me, but never on how the teachers working at my local junior high school experienced having a student like me, with cancer. I was made aware of this unexpectedly when talking to one of my former teachers. Even though our conversation was superficial at first—about the weather, my children, and his upcoming retirement—he then turned it to the years when my future was perceived as an abstract (even unlikely) entity. He talked about how he had gone out and bought a white linen cloth and a white candle, which he hid in a drawer in his classroom, while he mentally prepared to tell my friends and peers that I had passed. As I, in the hospital, fluctuated between dying and surviving for several months, he described this preparation and waiting as challenging. He concluded the subject by telling me that he later had bought several white linen cloths and candles and that he still had those first ones, which were meant for me. During this conversation, I suddenly became aware of myself. I started connecting personal memories and the professional present (Thoresen and Öhlén, 2015), and was overwhelmed by the thought that I could be one of those students carried around by teachers as I carry mine.

I wanted to rediscover myself as a teen and student with cancer. A search through the archives in my local municipality resulted in a slim folder with six documents, which offered some information. At the age of 15, I had been granted three hours of special needs education weekly to fill the academic gaps from my absence due to cancer. After assessments, it was concluded that I had profited from this. I can see that I have signed these documents, but I have no memories of doing so. In addition to these official documents, subjective (and sometimes ridiculously detailed and rawer) data can be found in my own diaries—written from the year of my diagnosis until I moved away from home at age 18—and in two full-to-bursting binders containing letters sent to me from my peers during my hospitalization(s).

In the diary I was writing when I was diagnosed, the often-contradictory experiences of both being very young and handling illness are richly illustrated. One day I list the boys in my class, assessing their behavior by giving them points. The next day I write four sentences: “I have leukemia. It is cancer. They will operate and put something into my vein, by the heart. The chemo will go in there.” After this, many weeks are left blank. During these weeks, letters from my friends fill in the gaps. In one letter, my best friend Maria writes both of a recent house party gone wrong, about how she misses my voice and presence, and how she prays to God, Allah, Buddha, and sometimes Satan to will me back to health and her everyday life. Through the emotions, memories, and conversations evoked while revisiting these artifacts, I start to write my narrative.

## The void of cancer kept pulling me back in

I have no photographs from when, at 13 and diagnosed with leukemia, I stayed for 54 straight weeks at Oslo University Hospital. During the first two weeks of treatments, my appearance changed beyond recognition. I did not look in mirrors. Cameras were prohibited. Although no photographic evidence exists, I have many memories from this period—some elusive, many vivid. The walls in my isolated room were a bright yellow. The clock in my room counted minutes, not seconds. From my bed, through the window, when lying down, I could see a rooftop. A crow had her nest there. I watched her bounce back and forth on the ridge.

I would look from the crow to the clock, visualizing what my friends were doing back home. At 08:15 am, Maria would arrive on the school bus. I would meet her at the bus stop. At 08:25 am, Kenneth, Ronny, and Fredrik would meet with Kine under the birch trees by the bike path. I would follow them into the schoolyard. At 08:30 am, the bell would ring, and mathematics, science, and the other subjects would follow. At around 11:06 am, Ronny would be reprimanded for flirting during class. I would hide my face behind my long blond hair. At 1:40 pm, Kenneth would look glassy-eyed out of the window. I would poke him with my pencil. At 3:15 pm, Kine would run to her bicycle and hurry to the stables. As she put the halter on her pony Caspar, I would hand her the heavy saddle. At 7:30 pm, Maria would be at home, curled up in a chair in her family’s living room, watching “Dawson’s Creek.” I was still lying in my hospital bed but also sitting in the chair next to her.

One of the bags with chemo—connected to my central vein catheter, hung to drip slowly into my veins—was covered with a pillowcase. Its color reminded me of tomato soup. I vomited so much coagulated blood that I thought I saw one of my inner organs in the paper kidney bowl. I shed my skin like a snake. My feet were on fire. My throat produced so much mucus that I could sit for hours, pulling it out through my mouth with my fingers. I was convinced it would suffocate me, but the nurses said it would not. The night before my organs failed, my eyelashes broke in half. The first time I had an epileptic seizure, I sensed it and said, “Now I am falling.” One of the nights when my O2 levels were low, I dreamed that the Earth had swallowed me and kept pulling me into its core. In the dream, I understood that I was inside the Earth. Looking up, I could see the faraway outside—a dim light coming from the Earth’s surface. From the inside of the Earth, I suddenly felt a sense of urgency. I realized I was late to school and started pushing through the Earth’s surface. When my arms or legs broke through, they bounced back as if a strong force was spiraling, pulling me inward and downward. When I woke up from the dream, I looked straight into the eyes of a nurse. She was holding a bag valve mask over my mouth and nose. Curiously, I noted that she looked afraid and that she was running beside my bed. I could tell that the pace was steady from the rapidly shifting fluorescent lights above my head. Too soon, I realized that we were heading in the direction of the ICU again. I knew this meant decline, that I had gotten worse, and that an unlimited amount of time waiting to get back to my life lay ahead. I thought of the bike path and the birch trees and Maria watching “Dawson’s Creek.” As the void of cancer kept spiraling me away from my peers, this was my heartache and my overwhelming grief.

## Learning cancer by doing chemo

I think of undergoing chemo as an extreme exercise in endurance. There really are no options. You swallow the pill. You let them poke, cut, tear, inject, extract, measure, and assess. There is no scheduled time for recess. Unconsciously, you train yourself—body and mind—to take it, obey, abide, endure, hang in, and push through. They mean well, and they want you to live, as they poison your body until it almost breaks. They pause. Wait for your vitals to stabilize. They continue. This is protocol. You thank them after every procedure.

I also think of cancer as an intense intellectual experience. I trained myself to pronounce words I had never heard. I contemplated first-time illness experiences as at first sensational, then matter-of-factly. How to keep a central vein catheter sterile, how to keep the dripline away from my father, who repeatedly got caught up in it, how many times to flush the toilet not to expose others to the poison in my body, what buttons to press on the infusion pump to mute it. My body parts in Latin, my brain, lungs, and blood through abbreviations and acronyms. The evolution of a cancerous cell, my leukocytes colored purple, seen through a microscope. I asked every adult that entered my room a myriad of questions: How come some get sick and others do not? Is this cancer the Devil’s work? Is it caused by sleeping near a high-voltage line? Or was it Chernobyl? Do you think I will die? What happens, exactly, when you die? I learned the oncologist’s tongue and shared hour-long philosophical conversations with my parents and the hospital priest.

Through diagnosis, I was first given a prognosis of 50% survival, and later no prognosis at all. It was communicated to my family, friends, and teachers that I was dying. I did not. However, I found a second home at the hospital, a 4-hour drive from my original one. In this second home, as a patient, I was primarily treated in the ICU. After, I was returned to a local hospital near my home and finally to my original home and local junior high school. Then, when the cancerous cells were no longer to be found in my body, I was considered cured. Besides the occasional blood work, a thorax x-ray, and a smear now and then, I was discharged and set free. After check-ups, I remember the nurses waving goodbye, saying jokingly, “Hope we’ll never see you again! Go get yourself a boyfriend!.” “Of course!” I would reply. “I will get several!.” I was meant to and wanted to grow up, but also out of cancer. However, despite well-intended encouragement, I found it hard to readjust to the healthy world awaiting me. The moratorium had not been lifted.

## The pre- and post-worlds of childhood cancer

Pre-cancer, I identified as an animal-loving, environmental activist, high-achieving, and popular pre-teen. I grew up in a house painted pink on an island in the south of Norway, where houses are traditionally painted white. My mother and father, both political activists—one a librarian, the other a teacher and plumber—had bookshelves in every room except the bathroom. My father, a wrestler, drove a yellow Lada he had painted himself with a roller. My mother, sharp as a knife and a tight budgeter, classified books as a general consumption expenditure. “You have a problem; you look for a book,” she said. My sister, five years older than me, taped “Friends” on our VHS recorder—one new episode was released weekly. She took time decorating each video cassette. I was seldom allowed to touch them, as she wanted to save them for her future children. I longed to wear Buffalo platform shoes and Adidas “Adibreak” pants with buttons from heel to hip but was not allowed. The summer before I was diagnosed, I had gained what I then considered the ultimate independence: I was allowed to go swimming with my friends in the ocean without adult supervision for the first time. When I jumped off the pier into the ocean that day, I distinctly remember being aware of the milestone that had been reached and feeling my growth as literal.

Post-cancer, I was both a tragedy and a miracle as “the girl who should have died.” While in the hospital, I suffered from both chemotherapy-induced polyneuropathy and critical illness syndrome, with acute paralysis, respiratory failure, multisystem organ failure, multiple inner bleeding, and sepsis. At its worst, I was unable to breathe without a ventilator, lost all my facial expressions, and was only able to move my neck and eyelids. When I began to heal, I could first sit upright in my bed only for a few minutes before vomiting furiously out of exhaustion and unfamiliarity. For a long time after, I was dependent on specialized health professionals and my parents to live and cope with day-to-day routines. Although people commented that my eyes were the same, I noticed that my physical changes—weight and hair loss, and muscular decay, especially—affected both children and adults. When interacting with me, they would suddenly seem at a loss, confused, occasionally turning away as if it hurt to look at me. Still, my persistent friends did their best to take care of me. They bravely pushed my wheelchair down very steep hills, visited me both in the hospital and at home or texted me from their recently achieved NOKIAs, when I was not fit to interact with them. Although often absent, as the whole local community knew my name and my burden, I was never forgotten. In this new life world, though, I was feeling stuck.

## The promises and perils of junior high school

Although now recently assessed as condemnable and torn down, I remember my local junior high school as an institution. Built in 1952 as a square, yellow brick building, its presence was one of impracticality, bad ventilation, and no heating. Twenty years ago, it was already outdated and worn down. Nevertheless, the building housed—at least in a new eighth grader’s opinion—many promises. Most often foregrounded was the promise of new flesh, as two different primary schools, placed a few miles apart, were merged into one. The anticipation projected onto the recently unknown boys coming from the east side of the island was stratospheric. In my school portrait from this period, I am confidently looking into the camera lens, wearing a blouse I had borrowed from my older sister without asking.

Built with three main floors, the school building did not have a functional elevator but steep and cold stone staircases: one in the middle and one on each side. Connected to these staircases, the building had three entryways. Perhaps naturally, the one in the middle was considered the main entrance. This was where most of the school’s 200 students entered and exited. I remember this as one large, shared movement, a sensory experience of being pushed into the backpack of the person in front of you or losing balance because of the driving forces of the masses from behind—in and upward after being called inside in the mornings, and downward and out after the last period had ended. A natural crossing point between grade levels and classes, this was a place to observe, be observed, and sometimes briefly touch.

When coming back to school after cancer, I entered through one of the side entrances. Unable to partake in activities at recess, I was confined to my classroom. There, my friends would take turns sitting with me, waiting for the next class to start. Sometimes we played cards, but most of the time we talked—about life and our everyday endeavors. One day, in confidence, a friend showed me a secret list: “The butts and boobs list” of the class. Scribbled on a crumbled piece of paper were the names of all the girls in the class but me. They were ranked by their appearance, from best to worst looking. “Where am I?” I asked while reading. “You?” he said. “*You* can’t be on that list.” My friend presented his answer as if it was a matter of courtesy. Pre-cancer, I’d only settle for a top-three ranking. After cancer, I’d be grateful for a bottom placement. Somehow, I had bounced off the list completely. I was in-between and undefined.

## “Today, I will carry your daughter”

Upon re-entry, my classroom was located on the third floor. It was only accessible by climbing those steep stone stairs. Then, I had regained enough strength to stand for a brief time while being supported or walking short distances indoors with leg braces and crutches. If not supported, I would fall hard to the ground, unable to catch myself. After falling, I would lie on the ground until someone mobilized the strength to lift me up and put me on my feet. The doctors considered me well enough to attend school, but my classroom was not physically available. I could not climb the stairs.

On the first day of re-entering, my father drove me to school and supported me into the school building. I remember standing at the bottom of the staircase—me, my father, a teacher, and some lingering peers—looking reluctantly at it. Upon realizing the obstacle at hand, my father acted seemingly without hesitation. “I have to lift her,” he said. Then, he placed one arm under my knees and one under my right arm and shifted my position from standing to lying in his arms. I remember someone trying to suggest other options, saying he should not climb all those stairs with me in his arms. Determined, almost as if he did not notice this, he started climbing, one step at a time, until he reached my classroom door and placed me in a chair inside the classroom.

To the memory of my father carrying my teenage self, no personal trauma is explicitly attached. In this period, I remember being carried around a lot, lying in my father’s arms like a small child. As an adult and a mother, I now look back and think about how this must have been excruciatingly hard for him, both physically and emotionally. His arms and back must have ached, and his heart was surely broken. However, together with my mother, he had been staying with me at the hospital, witnessing and experiencing my illness as if it was his own. When in hospital, he spent entire days singing old sea shanties in my ear or holding me, when in pain. My father was the strongest and steadiest man I knew. In his arms, I felt safe. Indirectly, though, being helplessly unable to enter my classroom without assistance, after always managing this before, was devastating.

I have no concept of how many times my father carried me up those stairs and into my classroom. However, I clearly remember the day this load was taken off him. A teacher—the same age as my father and looking quite similar to him—suddenly said, “Today, I will carry your daughter.” Perhaps surprisingly, my father trusted him enough to do so. As an adult and an educational counselor, I now think of this teacher and imagine how his feelings must have built up while watching my father’s efforts. I imagine his thoughts revolving around the simple notion of “a father should not have to carry his teenage daughter into her classroom,” and in addition, perhaps reflecting on how it could be experienced as challenging if he were to pursue it. Still, I have no prerequisites to understand what happened inside those two men before, during, and after this exchange. I can only remember that it happened and how it affected me.

While the teacher was carrying me up the stairs, I leaned into his chest. I remember the sensation of feeling another man’s arms around my body, how his muscles tensed, how his warmth transitioned from his body to mine, his unfamiliar but sweet smell, and the sound of his breath as he struggled to carry me. Until that point, I had taken my father’s strength for granted. With suspense, I wondered if this teacher would drop me out of exhaustion or destroy himself in the effort. He did not. But not long after, I started climbing the stairs on my own, with my hands clenching the railing, slowly pulling myself upward using my arms—one step at a time.

To this development, the teachers watched with dread, but my friends adjusted with ease. Fredrik would carry my crutches, and sometimes, Kine would support me while climbing. Then, Kenneth made sure he sat close enough so that I could poke him with my pencil without walking. Gradually, Ronny started flirting again. Slowly, we all maneuvered through the process of me coming back and becoming well. Sooner than expected, we grew up, and today, we talk about these experiences as something that has been surpassed. Nevertheless, my teacher’s involvement and effort that day dominate my perception of my re-entry. I have met him several times since but never asked him about it—although sometimes he tears up when he sees me.

## Afterthoughts

Goode (2019) writes that it is in the space between the experiencing self and the remembering self that we find ourselves, and Frank and Solbraekke (2023) describes remembering as iterative storytelling. By writing down my memories and asking others about theirs, I have awakened my illness narrative, and the narrative of becoming myself. My parents, my sister, my friends, my former teachers, and helpers—even though the cancer was in my body, they grew (up) with this too. This makes visible how my experiences are “tangled with others” (Adams, 2018: 203). It also makes visible how others serve as significant actors, both in narratives themselves but also in developmental- and illness trajectories.

I travel home in the summer. I stay for 2 weeks. I bring my partner, my two kids. I revisit the grounds where my junior high school once stood and find a part of the yellow brick wall and a part of the cold stone floor in the rubble. Almost obsessively, I jump again and again into the water from the same piers I jumped from as a teenager. In the few seconds I am underwater, I try to imagine myself at age 12, how my life was before cancer. Something has been triggered as I have been writing, re-writing, re-membering. I ask myself and others: When does an illness narrative end? Who should be given opportunities to voice their part? On what and on whose terms should it be voiced, and in what way must I write it to warrant its relevance as a scientific contribution?

I sit with my parents in their kitchen and tell them about my project. I start professionally and contained by telling them about the method, purpose, rationale, target journal, and the like. I look at them. My mom—an academic spirit, sharp, wise, also contained. My father, the wrestler, with kind eyes and heart in hand. Then, I ramble. My parents should have their retirement and live without worry or revisiting. I do not want to make my mother lose sleep, again, or re-traumatize my father. However, I need to write. I tell them, through sobs and snot and with a pitched voice, what I want and what I remember. How I recognize their struggles as an adult, as a parent myself. How I love them, how I am grateful for them, how my heart wishes they would not have experienced having a child like me, with cancer. They nod. When I stop crying, my mother responds, matter-of-factly: “First, there is no one to re-traumatize. This is our life. You can write these memories down, or you cannot—we carry them regardless.” My father does not object to this and slowly takes a sip of coffee.

I call my teacher. He is retired now and is about to go out for a swim with his wife. As with my parents, I tell him about my project and pursuit. I ask him if he remembers something from the time I was ill. At first, he is apologetic—he says he hopes schools solve these things differently today. Two memories, he says, are very clear. He was in charge of helping me apply for senior high school. I wanted to go to a school where the grade point average was high. There was some tension among the staff. Someone said out loud that perhaps I would not need an education or be able to complete one. Not everyone was driven by hope—they struggled to keep faith, he says. He remembers visiting my parents at home after work hours, finalizing the paperwork. He also describes visiting me in the hospital. I had asked for “my own teachers” and “real tutoring” in addition to the school activities arranged by the hospital school. He describes how I looked, at the hospital, sitting in bed. “Of course, I remember you, the little bird in the big white bed. You wanted to practice your French.,” he says.

I tell him about the lift. I present it as a tactile memory. “I was 14,” I say. “No man but my father had lifted me like that before.” Suddenly he goes quiet. Did he violate our teacher–student relationship? I elaborate and describe my memories as bittersweet yet core to my re-entry and narrative. I explain that I want insights into how he experienced this. Then he cries. After a while, he tells me his version of the lift. He talks about how he saw my parents’ exhaustion and my illness-ridden body, how he experienced differences in opinions and attitudes toward us, in staff, and between systems, structures, traditions. He wanted to stand up, he says. “I could handle this uncertainty. You did not scare me.” As we talk, I write down the words “uncertainty,” “frustration,” “flexibility,” “hope,” and “personal component.” We agree that we have started a strain of thought that will evolve.

## Gaining new insights from illness experience

In the Collins Online English Dictionary (Harper Collins, undated), re-entry is described as returning to somewhere one has left. It uses an example of a spacecraft re-entering Earth’s atmosphere after exploring space. In the educational sciences, re-entry refers to returning to a regular school setting after a prolonged absence. The school re-entry of a once familiar and healthy, now severely ill and changed teenager represents both a challenging process and a series of experiences. For the teenager, re-entry is a return and a change in location from hospital to school. When her status as a patient is finally replaced with the status of a student, her life horizon is made visible again. For the parents, re-entry is an exchange revolving around trust, control, and presence. As the father carries his daughter inside the classroom, unavailable to her, her participation is insisted upon. No guidelines are given to the teacher. While balancing educating and caring, he must mobilize from within, tolerate uncertainty, insist on hope, and dare to be bold. All the while, peers get glimpses into the interrelated worlds of health and disease, most for the first time. Despite the many efforts to support the re-entering student, re-entry is experienced as difficult all the same. Although not as rare as space travel, it poses as an extraordinary event holding lifelong influences.

The narrative made visible in this article illustrate how a bond can be created between a student and her teacher, led from situations deemed almost unresolvable. I asked my teacher about his memories from the time I was ill. Although all those years had passed, little was forgotten. While Adams and Bourke (2023) argue that teaching a student like the one I was, is a privilege and a potentially unique professional learning experience, I have learned that it can also represent strain and struggle. When I was re-entering, my teacher met me with a personal commitment and what he calls a personal component. Although my teacher acted intuitively and tells me he felt safe enough to do so, our shared vulnerability in this is evident. Presumably, this kind of vulnerability is inevitably evoked when a student suffers from severe illness and a teacher tries to accommodate her. Although very personal, these experiences make visible how illness transcends medicine. To accommodate them fully, collaborative and interdisciplinary efforts, and with that also efforts from the educational sciences, seem imperative.

## A new complimentary narrative type

Grant (2010) writes of autoethnographic ethics as the necessities and complications of (re)storying oneself. To the unvoiced void of childhood cancer, autoethnography feels like a timely but complicated trust exercise. Still, by being true to my own experiences and being attentive and open to others, my memories have found a form that should bear discussion and further examination. With this, my narrative enables me to “provide different imaginative conceptions of illness” (Frank, 2013: 187).

I try to match my illness narrative with Arthur Frank’s narrative types and to align my lived experience with them. As I was provided with state-of-the-art treatment and looked forward to regaining my old self, I lived in a restitution narrative. As my health declined and my healing was delayed, my illness represented chaos. As I learned to speak the oncologist’s tongue, my illness evolved into an intellectual challenge similar to quest. Still, I cannot shake the feeling of these analytical frames of thinking falling short of what I perceive as central to my embodied experience, the formative phase I was amid.

My narrative presents illness as something you grow up with. When trying to pin down the traits of my story, I think of the stark contrasts between the inevitable pace and forwardness of growing up and the excruciating slow pace imposed by the many, sometimes rapidly shifting stressors of illness. I also think of the lingering and dominating feeling of being confused and out of place and the prolonged interval when the outcome of my struggles was perceived as unclear. For a long time, I did not experience a coherent sense of self or find a workable adjustment to my cancer. When pondering this, I catch myself thinking “if only”: If only my sense of self was more fixed, if only I had found the others I now relate to as my own, if only I had established a clear vision of myself in the future, my illness narrative would have been fundamentally different. As Frank (2000) explains that his work with illness narratives was led from an experienced line of fault, a mismatch between lived experience and academic descriptions, I seem to have experienced my own.

In my narrative, experiences related to being very young and ill share a tug of war. Here, neuropathy and Christmas proms, liver monitoring, and illegal drinking, simultaneously contemplating the formula of square meters and the purpose of pain, experiencing my first kiss, and enduring my friend’s funeral, go hand in hand. By far, most of these experiences seem to share their own narrative characteristics, have no designated place in the narrative of childhood cancer, nor have been provided a suitable narrative type. As the confusing conflicts of the yet-to-be-solved defined my developmental trajectory and my illness trajectory and now define my illness narrative, I find the need to propose a fresh narrative resource. I name this the moratorium narrative.

As with the term liminality, moratorium could be seen as a “rubric of a category” (Little et al., 1998: 1485). As such, with the means of the moratorium, the specific experiences related to being young and very ill can be interpreted for both practical and theoretical purposes. As a fresh resource, it can make sensitive our academic community’s gaze and help our scientific community recognize illness trajectories unfolding in formative phases and illness narratives defined by growing up. Ideally, it can also support the writer, the reader, and the thinker, when acquired to hold a split focus on youth as an existential expectation and illness as an existential threat.

Adding to the momentous inquiry of illness, the moratorium narrative should honor the undecided length of time navigated when simultaneously adjusting to or negotiating illness and growing up. In this, the well-known identity struggles, the pressures and expectations, and the promises and perils related to both clusters of experiences should be equally recognized. As such, although derived from a specific theory on development, the moratorium narrative provides a new perspective on the embodiment of illness and holds multidisciplinary potential. Hopefully, it also provides a point of recognition that prompts elaboration, countering, and remaking. Ideally, to the young and very ill, it will provide a much-needed narrative space of opportunity, of which more narratives are invited and insisted upon. However, in the end, its utility will prove itself and evolve with writers answering its call.
